# Evaluation of satisfaction with care in a midwifery unit and an obstetric unit: a randomized controlled trial of low-risk women

**DOI:** 10.1186/s12884-016-0932-x

**Published:** 2016-06-18

**Authors:** Stine Bernitz, Pål Øian, Leiv Sandvik, Ellen Blix

**Affiliations:** Department of Obstetrics and Gynecology, Østfold Hospital Trust, Sarpsborg, Norway; Department of Obstetrics and Gynecology, the University Hospital of North Norway, Tromsø, Norway; Department of Clinical Medicine, Faculty of Health Sciences, University of Tromsø, N-9037 Tromsø, Norway; Unit for Biostatistics and Epidemiology, Oslo University Hospital, Oslo, Norway; Faculty of Dentistry, University of Oslo, Oslo, Norway; Faculty of Health, Oslo and Akershus University College of Applied Sciences, Oslo, Norway

**Keywords:** Low-risk birth, Intrapartum care, Satisfaction, Birth care unit

## Abstract

**Background:**

Satisfaction with birth care is part of quality assessment of care. The aim of this study was to investigate possible differences in satisfaction with intrapartum care among low-risk women, randomized to a midwifery unit or to an obstetric unit within the same hospital.

**Methods:**

Randomized controlled trial conducted at the Department of Obstetrics and Gynecology, Østfold Hospital Trust, Norway. A total of 485 women with no expressed preference for level of birth care, assessed to be at low-risk at onset of spontaneous labor were included. To assess the overall satisfaction with intrapartum care, the Labour and Delivery Satisfaction Index (LADSI) questionnaire, was sent to the participants 6 months after birth. To assess women’s experience with intrapartum transfer, four additional items were added. In addition, we tested the effects of the following aspects on satisfaction; obstetrician involved, intrapartum transfer from the midwifery unit to the obstetric unit during labor, mode of delivery and epidural analgesia.

**Results:**

Women randomized to the midwifery unit were significantly more satisfied with intrapartum care than those randomized to the obstetric unit (183 versus 176 of maximum 204 scoring points, mean difference 7.2, *p* = 0.002). No difference was found between the units for women who had an obstetrician involved during labor or delivery and who answered four additional questions on this aspect (mean item score 4.0 at the midwifery unit vs 4.3 at the obstetric unit, *p* = 0.3). Intrapartum transfer from the midwifery unit to an obstetric unit, operative delivery and epidurals influenced the level of overall satisfaction in a negative direction regardless of allocated unit (*p* < 0.001).

**Conclusion:**

Low-risk women with no expressed preference for level of birth care were more satisfied if allocated to the midwifery unit compared to the obstetric unit.

**Trial registration:**

The trial is registered at www.clinicaltrials.govNCT00857129. Initially released 03/05/2009.

## Background

Low-risk birth care units intend to present an alternative setting for women with low risk of complications during labor. These birth units are established to counterbalance the trend of centralizing birth care in obstetric units that has been occurring over recent decades in industrialized countries. The design of the low-risk birth care units is often a homelike environment with medical and technical equipment on a low or a moderate level and a philosophy committed to the normality of childbirth [1]. The Department of Obstetrics and Gynecology at Østfold Hospital Trust is one of five large obstetric clinics in Norway which have established alongside midwifery units. These units aim to provide an alternative setting for birth for women with low risk of complications who regard labor and delivery as a physiological process.

Medical outcomes in low-risk birth care units have been evaluated both in randomized controlled trials [1] and in cohort studies [2–4]. The trials show that women laboring in low-risk birth care units have a reduced risk of interventions like epidural analgesia, augmentation with oxytocin [1–3, 5], and a reduced risk of operative deliveries [2, 3] without jeopardizing the outcome for the mother or the baby [1–3]. The economic perspective of laboring in low-risk birth care units compared to obstetric units for low-risk women has also been investigated, revealing both cost savings and improved outcomes [6, 7].

When evaluating low-risk birth care units it is crucial that patient satisfaction is included as a quality indicator in addition to health related outcomes [8] to provide a user oriented focus. Of ten studies included in a Cochrane review on alternative versus conventional institutional settings [1], five studies from Sweden, UK and Australia presented satisfaction with care [9–13]. In three of the studies women could choose to participate in the trials or choose to refrain and deliver at the unit they preferred [9–11]. In the other two studies women who wanted to deliver in the alternative setting had to be enrolled in the trial [12, 13]. The review concluded that birth care units were associated with higher levels of satisfaction. A Swedish cohort compared birth center care with standard maternity care, and found that the odds for being satisfied overall were approximately doubled in the birth center group [14]. In the NICE clinical guideline for intrapartum care, it is found that if there are differences between the groups, women giving birth in low-risk units, either alongside or freestanding, are more satisfied compared to laboring in obstetric units. Still the included studies on satisfaction are few and of varying quality; hence there are few randomized controlled trials on satisfaction with birth care [8].

Satisfaction with birth care is found to be influenced by several factors. The significance of the birthing environment is shown by the fact that women giving birth in alternative settings with a homelike environment express greater satisfaction than women giving birth in obstetric units [1]. The feeling of support and the relationship between the woman and the caregiver are factors with major impact on the level of satisfaction found both in qualitative research [15, 16] and in quantitative research [17–20]. Active involvement in decision making processes fosters a sense of empowerment and self-efficacy [17] and is among factors women emphasize when assessing satisfaction with labor care [17–20]. The effect of labor pain on satisfaction with birth care is subordinate compared to the interpersonal aspects mentioned above [19, 21]. Continuity of care has also been proven to be of importance when assessing women’s satisfaction [22–24].

The main aim of this trial was to investigate possible differences in satisfaction with intrapartum care in low risk-women randomized to a midwifery unit and to obstetric units within the same hospital in Norway. Specific factors influencing the level of satisfaction were not investigated.

## Methods

### Organization of care services

The Department of Obstetrics and Gynecology at Østfold Hospital Trust is divided in three separate birth care units within the same hospital building, the midwifery unit (MU), the normal unit (NU) and the special unit (SU). The MU is organized for women who want a normal labor without interventions and who fulfill the following criteria: healthy, low-risk of complications, single vertex infant, pre-pregnant Body Mass Index (BMI) ≤ 32, not smoking more than 10 cigarettes per day, no prior operation on the uterus, no prior complicated delivery and spontaneous onset of labor between gestational weeks 36^+1^ and 41^+6.^ The unit does not offer epidural analgesia nor augmentation with oxytocin. Obstetricians are not present at the MU, but will come when called for. Women who need extended surveillance or medical pain relief are transferred to the NU or the SU. The NU is organized for women with expected normal labor, but the unit offer extended surveillance, epidural analgesia and instrumental vaginal deliveries. The SU offers women with a need of extended surveillance the necessary monitoring and treatment throughout pregnancy, labor and delivery and after birth. Subject to capacity low-risk women are welcome at any of the three units. All units provide labor, delivery and postpartum care. The NU and the SU combined is considered an obstetric unit and hereafter named the obstetric unit (OU). Antenatal care is provided by midwives in primary care; hence no continuity of care is offered at the midwifery unit nor at the obstetric unit.

### A randomized trial

From 2006 to 2010 a randomized controlled trial was carried out at the department to investigate possible differences in outcomes, including satisfaction with intrapartum care, among low-risk women, randomized to the MU, NU or to the SU [25]. The results showed a non-significant difference in operative delivery rate, but revealed that low-risk women allocated to the MU had a significant higher chance of giving birth without interventions like epidural analgesia and augmentation with oxytocin without affecting the outcome for mother and baby compared to the OU [25].

All participants had to fulfill the inclusion criteria which were similar to the selection criteria at the MU. Women considered to be low-risk and who wanted to participate were recruited by signing an informed consent at the ultrasound consultation. If they were still eligible at onset of spontaneous labor they were randomized to one of the three units to ensure that every participant was considered low-risk when included. Of 2884 eligible women at 18 weeks of gestation, 1111 were still eligible and willing to participate at onset of spontaneous labor. The randomization process was conducted through an electronic program stratifying for parity and with concealed allocation. Due to capacity challenges, randomization was pre-specified to allocate 37.5, 37.5 and 25.0 % to the MU, NU and SU, respectively.

### Satisfaction with intrapartum care

A secondary outcome of the randomized controlled trial was satisfaction with intrapartum care and we wanted to investigate possible differences in women’s satisfaction between the units using the Labour and Delivery Satisfaction Index (LADSI) [26]. LADSI is a validated 38-item questionnaire measuring “technical” and “caring” components of satisfaction [27]. Each item in the questionnaire was worded as a statement for ratings of agreement or disagreement on a six-point Likert scale. The items in the questionnaire were created on the basis of a literature review and interviews with new mothers in addition to clinical experience [27]. The questionnaire has face validity and content validity, but due to less than optimal internal consistency, the developers recommend that LADSI only be presented in total [28]. All participants answered the first 34 questions concerning overall satisfaction with birth care. Due to the fact that midwives in Norway are responsible for normal labors and deliveries, and that obstetricians are involved only in deliveries requiring obstetric support, the last four items of the LADSI were only answered by women who had an obstetrician involved in her labor and/or delivery as these were questions about doctors involvement.

As we investigated low-risk women in different birth settings, we also wanted to investigate women’s satisfaction with intrapartum transfer from the MU to the OU. To explore the latter outcome, four items were added to the questionnaire in a separate box to be answered only by women who were transferred from the MU during labor or delivery.

The measurement of satisfaction is especially challenging in the perinatal period and the individual response may be affected by changing moods [17]. There is a tendency of higher levels of satisfaction close in time to birth [28] and declining levels over time up to 2 years [21]. In a systematic review by Hodnett, 34 studies on satisfaction with intrapartum care were included with a range of timing from immediately after birth up to one year post partum [19]. In this study the questionnaire was sent 6 months after birth to minimize altered post partum moods and still in reasonable time according to completion of the trial.

**Fig. 1:**
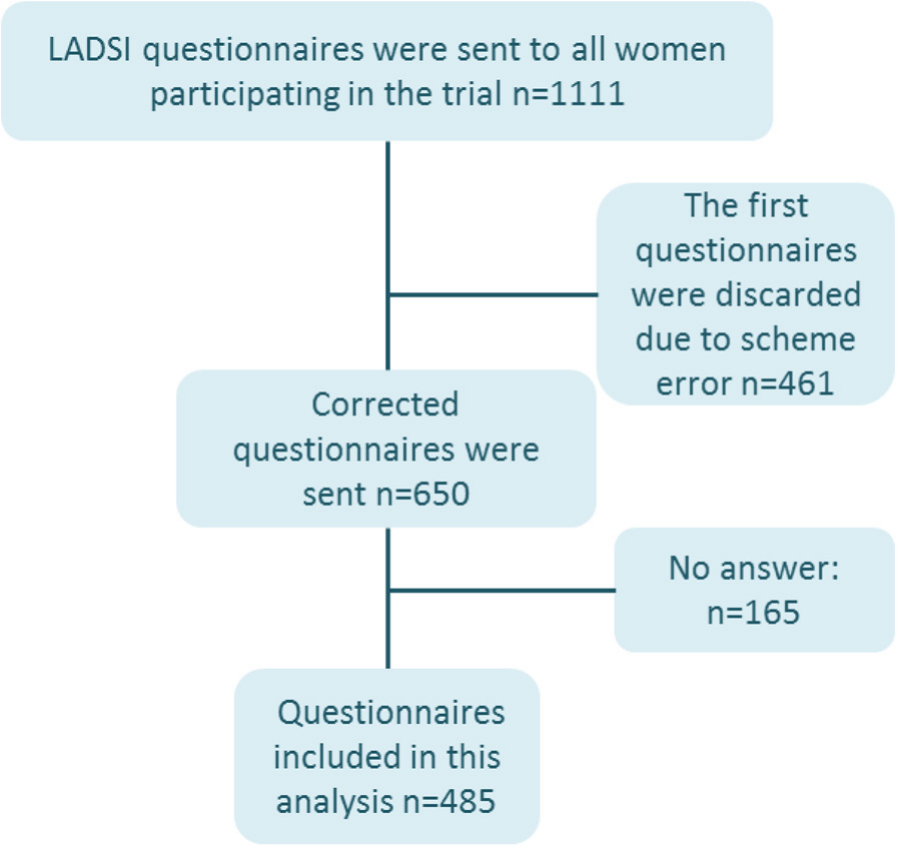
Flowchart of inclusion

The questionnaires were sent, in stamped and addressed envelopes to all women participating in the randomized controlled trial. Written reminders were sent to non-responders 4 weeks after distribution. A partway through the study period, an error in the Likert scale point numbers on the printed questionnaires (five instead of six points) was discovered and the first 461 distributed questionnaires were discarded due to this error. The error was corrected and revised and correct questionnaires with a six points Likert scale were sent to participants included in the study (650) from November 2007 to March 2010 (Fig. 1).

The 461 women who received the questionnaires with an error, did not differ in characteristics or outcomes from those included in the analyses. Thus, the mechanism is “missing complete at random”, meaning that it is unrelated to anything inferred from the data [29].

The questionnaires were optically scanned by direct transfer of data from a written form to an electronic file. We used the program Teleform, Cardiff-Software version 7, to design and scan the forms after data collection. The program compares the written data in the form with what is expected from the specifications for each field in the form. The data were then transferred to a SPSS file. The data file was controlled by a research consultant who was blinded to the participants’ trial arm.

### Statistical analysis

Power calculations were conducted for the randomized controlled trial on the main outcome; cesarean section, power calculations on satisfaction in this paper were therefore conducted post hoc.

In the MU, *n* = 184 and in the OU *n* = 301, the standard deviation of sum score was close to 25 in each group. When comparing the groups, an independent samples *t*-test was applied, with 5 % significance level. We consider the difference in mean Sum score between the groups to be of clinical importance when it is at least 7. It may be shown that when the true difference in mean Sum score between the groups is at least 7, the test power in the present study is at least 95 %. Thus our study appears to have an adequate test power.

Outcomes are descriptively presented in numbers (Table 2). To detect if there were differences between the units in satisfaction with intrapartum care, scores were compared using an independent samples *t*-test. Results are presented in mean scores with standard deviation, differences between the units are presented in mean difference with a 95 % Confidence Interval (95 % CI) and in p-values (Table 3).

To explore if interventions like epidural analgesia and operative delivery, or obstetricians’ involvement affected the level of satisfaction, analyses were conducted according to outcome.

Assuming that mode of delivery affected level of satisfaction after being transferred from the MU to the OU intrapartum, or after an obstetrician was involved, subgroup analyses were conducted. Results are presented in mean scores with standard deviation, differences according to outcome are presented in mean difference with a 95 % Confidence Interval (95 % CI) and in p-values (Table 4).

To explore women’s experience with intrapartum transfer, scores of the four additional items on transfer are presented descriptively in mean score for each question with standard deviation as only women randomized to the MU were transferred intrapartum (Table 5).

The analyses were conducted according to the principle of intention-to-treat. Missing values (<1 %) were replaced by imputation. All statistical analyses were conducted using Statistical Product and Service Solutions (SPSS) version 18.

## Results

The questionnaire was sent to 650 women of whom 485 responded (74.6 %). Of the 244 women randomized to the MU, 184 responded (75.7 %) and of the 406 women randomized to the OU, 301 responded (74.1 %), *p* = 0.657. The basic characteristics of all participants were similar between the compared groups (Table 1). Of all included women, 70.1 % were nulliparous, 52.6 % had more than 13 years of education, 64.5 % were aged between 25 and 35 years and 95.9 % were married or cohabiting. The number of women who had an obstetrician involved during labor or delivery was 37 (20.1 %) at the MU and 78 (25.9 %) at the OU, *p* = 0.154. The number of women who had an operative delivery was 25 (13.6 %) at the MU and 65 (21.6 %) at the OU, *p* = 0.028. For epidurals the numbers were 25 (13.6 %) at the MU and 68 (22.6 %) at OU, *p* = 0.015. Of all 184 women who were randomized to the MU, 53 (28.8 %) were transferred to the OU during labor or delivery (Table 2).

**Table 1:** Basic characteristics of the participants

Birth Unit	MU^a^	OU^b^	All	p-value
	*n* = 184 (%)	*n* = 301 (%)	*n* = 485 (%)	
Parity				
Nulliparous	129 (70.1)	214 (71.1)	343 (70.7)	
Multiparous	55 (29.9)	87 (28.9)	142 (29.3)	0.817
Education				
≤ 13 years	84 (45.7)	141 (46.8)	225 (46.4)	
> 13 years	97 (52.7)	158 (52.5)	255 (52.6)	
Missing	3 (1.6)	2 (0.7)	5 (1.0)	0.874
Age				
< 25 years	37 (20.1)	75 (24.9)	112 (23.1)	
25–35 years	126 (68.5)	187 (62.1)	313 (64.5)	
> 35 years	21 (11.4)	39 (13.0)	60 (12.4)	0.522
Marital status				
Married/Cohabitant	175 (95.1)	290 (96.3)	465 (95.9)	
Single	9 (4.9)	11 (3.7)	20 (4.1)	0.507

^a^Midwifery Unit

^b^Obstetric Unit

**Table 2:** Outcomes and interventions according to birth care unit

	MU^a^	OU^c^	All	*p*-value
	*n* = 184 (%)	*n* = 301 (%)	*N* = 485 (%)	
Obstetrician involved	37 (20.1)	78 (25.9)	115 (23.7)	0.154
Operative delivery^c^	25 (13.6)	65 (21.6)	90 (18.6)	0.028
Epidural analgesia	25 (13.6)	68 (22.6)	93 (19.2)	0.015
Intrapartum transfer	53 (28.8)	-	-	-

^a^Midwifery Unit

^b^Obstetric Unit

^c^Cesarean section, assisted vaginal delivery

For the first 34 items concerning overall satisfaction with intrapartum care, the maximum score was 204. Of all participants, those randomized to the MU had significant higher mean score (182.7) than those randomized to the OU (175.5) total mean score difference 7.2, CI 95 % 2.6–11.8, *p* = 0.002. Of women with spontaneous deliveries, those randomized to the MU had a mean score of 187.0, and those randomized to the OU had a mean score of 180.0, mean score difference 6.8, CI 95 % 2.2–11.4, *p* = 0.004. Of women with spontaneous deliveries and no interventions, those randomized to the MU had a mean score of 190.7 and those randomized to the OU had a mean score of 183.2, mean score difference 7.5, CI 95 % 2.9–12.1, *p* = 0.001 (Table 3).

**Table 3:** Overall satisfaction with intrapartum care according to unit, using the Labour and Delivery Satisfaction Index (LADSI) with a maximum score of 204

	MU^a^	OU^b^	Mean difference (95 % CI)	*P*-value
	*n* = 184	*n* = 301		
	Mean score (SD)	Mean score (SD)		
All (*n* = 485)	182.7 (23.3)	175.5 (27.7)	7.2 (2.6–11.8)	0.002
Obstetrician involved	160.4 (25.7)	161.6 (29.8)	−1.26 (−12.6–10.0)	0.825
Operative delivery^c^	155.1 (28.7)	158.3 (29.9)	3.2 (−16.9–10.0)	0.649
Epidural analgesia	155.6 (29.5)	163.6 (32.7)	8.0 (−22.9–6.8)	0.285
Spontaneous delivery	187.0 (19.1)	180.2 (25.1)	6.8 (2.2–11.4)	0.004
Spontaneous delivery, no intervention^d^	190.7 (16.3)	183.2 (22.5)	7.5 (2.9–12.1)	0.001
Intrapartum transfer	162.5 (26.0)	-	-	-

^a^Midwifery Unit

^b^Obstetric Unit

^c^Cesarean section, assisted vaginal delivery

^d^No transfer, no obstetrician involved, no epidural

The 115 (23.7 %) women who had an obstetrician involved during labor or delivery answered the four last items of the LADSI. There were no differences in mean scores for overall satisfaction with intrapartum care between women who had an obstetrician involved at the MU 160.4 or at the OU 161.6, mean difference −1.3, CI 95 % −12.6–10.0, *p* = 0.825. (Table 3). Nevertheless, women who had an obstetrician involved, expressed significant lower overall satisfaction with intrapartum care 161.2, than those who did not 183.5, mean difference 22.3, CI 95 % 17.1–27.4, *p* < 0.001 (Table 4). Assuming that mode of delivery where an obstetrician was involved affected the level of satisfaction, we conducted an analysis comparing overall satisfaction with intrapartum care among all women who had an obstetrician involved according to operative delivery. Women who had an obstetrician involved and had an operative delivery had a mean score for overall satisfaction with intrapartum care of 154.9. Women who had an obstetrician involved and had a spontaneous vaginal delivery had a mean score for overall satisfaction with intrapartum care of 171.0, mean difference 16.1, CI 95 % 6.1–26.1, *p* = 0.002 (Table 4).

**Table 4:** Overall satisfaction with intrapartum care according to outcome, using the Labour and Delivery Satisfaction Index (LADSI) with a maximum score of 204

Outcome	Yes mean score (SD)	No mean score (SD)	Mean difference (95 % CI)	*p*-value
Obstetrician involved	161.2 (28.5)	183.5 (23.2)	22.3 (17.1–27.4)	<0.001
Operative delivery^a^	157.4 (29.4)	183.0 (23.1)	25.5 (19.0–32.1)	<0.001
Epidural analgesia	161.5 (32.0)	182.2 (23.1)	20.7 (13.8–27.7)	<0.001
Intrapartum transfer	162.5 (26.0)	190.9 (16.2)	28.4 (20.7–36.0)	<0.001
Operative delivery^a^ if obstetrician involved	154.9 (29.3)	171.0 (24.4)	16.1 (6.1–26.1)	0.002
Operative delivery^a^ if transferred intrapartum	151.4 (26.7)	171.0 (22.4)	19.7 (5.8–33.6)	0.007

^a^Cesarean section, assisted vaginal delivery

### Satisfaction with intrapartum transfer

The 53 women (28.8 %) who were transferred from the MU to the OU during labor or delivery answered four additional items concerning satisfaction with intrapartum transfer. The four additional items were: 1. I was satisfied before transfer, mean item score 4.96, 2. I was satisfied after transfer, mean item score 4.70, 3. The transfer felt like a burden, mean item score 3.68 and 4. I felt involved in the process of transfer mean item score 3.94 (Table 5).

**Table 5:** Satisfaction with intrapartum transfer from MU to OU *n* = 53. Four additional items with a maximum score of 6 points per item

`	mean score (SD)
Satisfied before the transfer	4.96 (1.57)
Satisfied after the transfer	4.70 (1.61)
The transfer felt like a burden	3.68 (2.05)
Felt involved in the transfer	3.94 (1.62)

The mean score for overall satisfaction with intrapartum care was 162.5 for women who were transferred during labor or delivery compared to 190.9 for those who stayed in the MU throughout labor and delivery, mean difference 28.4, CI 95 % 20.7–36.0, *p* < 0.001 (Table 4).

Women who were transferred intrapartum and had an operative delivery had a mean score for overall satisfaction with intrapartum care of 151.4. Women who were transferred intrapartum and had a spontaneous vaginal delivery had a mean score for overall satisfaction with intrapartum care of 171.0, mean difference 19.7, CI 95 % 5.8–33.6, *p* = 0.007 (Table 4).

### Subgroup analyses

There was no difference in mean score for overall satisfaction with intrapartum care between the units for women who had an operative delivery 155.1 compared to 158.3 at the MU and OU respectively, mean difference 3.2, CI 95 % −10.7–17.0, *p* = 0.649. For women who had an epidural there was no difference in mean score for overall satisfaction with intrapartum care between the MU 155.6 and the OU 163.6, mean difference 8.0, CI 95 % −6.8–22.9, *p* = 0.285 (Table 3).

The two interventions, operative delivery and epidurals had an influence on the rate of mean score for overall satisfaction with intrapartum care without differing between the units. The mean score for intrapartum care for women with an operative delivery was 157.4 compared to women with a spontaneous vaginal delivery 183.0 mean difference 25.5, CI 95 % 13.8–27.7, *p* < 0.001. The mean score for overall satisfaction with intrapartum care for women with an epidural was 161.5 compared to 182.2 for women without epidurals, mean difference 20.7 CI 95 % 13.8–27.7, *p* < 0.001 (Table 4).

## Discussion

Satisfaction with intrapartum care is multidimensional, dynamic and influenced by a variety of different factors [17]. Socio-demographic factors have been found to influence the level of satisfaction. Lower levels of satisfaction are shown to be associated with low level of education [17], young age, single status and primiparity [17, 20]. In our trial the characteristics of the participants were equal between the compared groups due to the design; hence we did not investigate the significance of socio-demographic factors.

In this trial we aimed to investigate if level of birth care had any influence on satisfaction with care for low-risk women, and we found that the overall satisfaction with intrapartum care was significantly higher among women randomized to the MU compared to the OU. The clinical relevance of the difference in mean score is unclear as one could argue that it is small. The LADSI does not recommend a cut-off for a clinical meaningful difference. Prior research is in accordance with our findings and has shown that women in midwifery units or birth centers are more satisfied than women in obstetric units [13, 14, 28] and that women laboring in alternative settings for birth are more satisfied than women in standard settings [14]. As the LADSI does not have items directly pointing towards the environment or the setting, conclusions on the significance of the environment cannot be drawn in our trial.

For women who had an obstetrician involved during labor or delivery in our study, group affiliation did not affect the assessment of satisfaction. Nevertheless women who had an obstetrician involved were less satisfied than those who did not. We did not investigate reasons for obstetricians’ involvement and reasons for dissatisfaction in details. However, we might assume that for low-risk women with expected normal deliveries, the fact that an obstetrician needed to be consulted in itself could be more important than the actual meeting with the obstetrician [30].

Giving birth in a low-risk birth care unit implies the risk of being transferred to an obstetric unit intrapartum if extended surveillance is needed, or if the birth needs to be handled by an obstetrician. The impact of intrapartum transfer on satisfaction with birth care from a freestanding birth care unit or from an alongside unit to an obstetric unit is not well known. Even though women who were transferred intrapartum in our study were less satisfied than those who did not, the overall satisfaction were higher among women randomized to the MU compared to those randomized to the OU. When analyzing the difference in overall satisfaction with intrapartum care for women being transferred intrapartum, our findings might indicate that both an operative delivery as well as an intrapartum transfer in itself play an important role for satisfaction.

As we assumed that some outcomes could affect the feeling of satisfaction, two subgroup analyzes were conducted; mode of delivery and epidural analgesia. We found that women with an operative delivery, either emergency cesarean section or instrumental vaginal delivery were less satisfied than women who delivered spontaneously. Our finding do not concur with a study by Spaich et al. [31] as they found that mode of delivery did not affect the level of satisfaction for low-risk women. Women who had an epidural for pain relief were significantly less satisfied than those who did not have an epidural in our trial. A Cochrane review on the use of epidurals found on the other hand, no significant difference in maternal satisfaction with pain relief between those who had an epidural and those who did not [32]. The Cochrane review investigated satisfaction with pain relief and not overall satisfaction with intrapartum care. Neither of the two factors mentioned above influenced the rate of satisfaction between the units.

Evaluating satisfaction with birth care is challenging, of all 11 included trials in a Cochrane review on midwifery units versus other models of care for childbearing women, nine included satisfaction with care. However, due to inconsistency in the instruments, scales, timing of administration and outcomes used to measure satisfaction, a meta-analysis was not conducted. Still it was concluded that satisfaction in various aspects of care appeared to be higher in the midwifery units compared to other models of care [23].

It is worth noting that all participants included in this study had no expressed preference for level of birth care. Conclusions can therefore only be drawn for women without conscious choice of birth place. As the LADSI is recommended to be presented in total, specific factors influencing the level of satisfaction will not be revealed in this trial. Despite this, women randomized to the MU were overall more satisfied compared to women randomized to the OU regardless of interventions and intrapartum transfer.

The specific reasons why women randomized to the MU are overall more satisfied cannot be confirmed in this study. It might be influenced by a combination of the homelike environment, the philosophy of the staff or may be the fact that the activity level is calmer due to women’s low-risk status.

## Conclusions

The significantly higher satisfaction score of women randomized to the midwifery unit together with good clinical outcomes, suggest that birth care units is a preferable option for low-risk women.

### Abbreviations

LADSI, The Labour and Delivery Satisfaction Index; MU, midwifery unit; NU, normal unit; OU, obstetric unit; SU, special unit
